# GPR3 Receptor, a Novel Actor in the Emotional-Like Responses

**DOI:** 10.1371/journal.pone.0004704

**Published:** 2009-03-04

**Authors:** Olga Valverde, Evelyne Célérier, Mária Baranyi, Pierre Vanderhaeghen, Rafael Maldonado, Beata Sperlagh, Gilbert Vassart, Catherine Ledent

**Affiliations:** 1 Grup de Recerca de Neurobiologia del Comportament, Departament de Ciències Experimentals i de la Salut, Universitat Pompeu Fabra, Barcelona, Spain; 2 Laboratori de Neurofarmacologia, Departament de Ciències Experimentals i de la Salut, Universitat Pompeu Fabra, Barcelona, Spain; 3 Institute of Experimental Medicine, Hungarian Academy of Sciences, Budapest, Hungary; 4 IRIBHM, Université Libre de Bruxelles (ULB), Campus Erasme, Brussels, Belgium; Pennsylvania State University, United States of America

## Abstract

GPR3 is an orphan G protein-coupled receptor endowed with constitutive Gs signaling activity, which is expressed broadly in the central nervous system, with maximal expression in the habenula. We investigated the consequences of its genetic deletion in several behavioral paradigms and on neurotransmission. Compared to wild-type, hippocampal neurons from *Gpr3^−/−^* mice displayed lower basal intracellular cAMP levels, consistent with the strong constitutive activity of GPR3 in transiently transfected cells. Behavioral analyses revealed that *Gpr3^−/−^* mice exhibited a high level of avoidance of novel and unfamiliar environment, associated with increased stress reactivity in behavioral despair paradigms and aggressive behavior in the resident-intruder test. On the contrary, no deficit was found in the learning ability to avoid an aversive event in active avoidance task. The reduced ability of *Gpr3* ^−/−^ mice to cope with stress was unrelated to dysfunction of the hypothalamic-pituitary-adrenal axis, with *Gpr3^−/−^* mice showing normal corticosterone production under basal or stressful conditions. In contrast, dramatic alterations of monoamine contents were found in hippocampus, hypothalamus and frontal cortex of *Gpr3^−/−^* mice. Our results establish a link between tonic stimulation of the cAMP signaling pathway by GPR3 and control of neurotransmission by monoamines throughout the forebrain. GPR3 qualifies as a new player in the modulation of behavioral responses to stress and constitutes a novel promising pharmacological target for treatment of emotional disorders.

## Introduction

How signal transduction pathways control different aspects of behavior is a central issue in neurobiology. Recently, the cAMP signaling pathway has been implicated in the action of chronically administrated antidepressant drugs [1]. In addition, alterations in the cAMP pathways have been reported to produce major disturbances in emotional behaviors [2], [3], [4], [5]. Here, we examine the implication of GPR3, a novel potentially important regulator of intraneuronal cAMP, in the modulation of emotional behavior.

GPR3 is an orphan G-protein-coupled receptor (GPCR) which, upon transfection in various mammalian cell lines, causes strong constitutive activation of adenylyl cyclase, in the absence of any added agonist [6]. In mouse oocytes, GPR3 contributes to maintenance of cAMP concentrations at a level required to ensure meiotic arrest in prophase I until the LH surge [7], [8], [9]. Whether cAMP accumulation is the result of a true constitutive activity of the receptor or the consequence of the chronic stimulation by a ubiquitous unknown ligand, is still debated. Sphingosine 1-phoshate was proposed as an agonist of the rat GPR3 homologue [10], [11] but this has not been confirmed yet.

GPR3 transcripts are also widely expressed in the mouse brain, in areas related to different physiological functions. More specifically, GPR3 receptor is expressed in the main brain structures involved in stress-related behaviors such as habenula but also hippocampus, amygdala, limbic system and cortex. Interestingly, the highest levels of expression were found in the habenula. The habenular complex is an important relay station between the limbic forebrain and the midbrain. It has been clearly shown to participate in the regulation of ascending monoamine and acetylcholine transmission towards hippocampus and frontal cortex [12], [13]. Serotonin and noradrenaline systems are known to play a role in the regulation of several central activities including mood and anxiety. Dysregulation of these systems appears to have a role in the pathophysiology of depression and anxiety disorders [14], [15]. Given the ability of GPR3 to activate the cAMP regulatory cascade in a tonic way in areas involved in stress-related behaviors, we hypothesized that GPR3 could play a role in the control of the corresponding behavioral responses.

In the present study, we have directly addressed this issue by analyzing the behavioral phenotype of *Gpr3^−/−^* mice generated by homologous recombination, focusing on the evaluation of responses related to emotional behavior. The relevance of this new system in the stress-related responses was evaluated in the hormonal modulation of the hypothalamic-pituitary adrenal axis and the monoamines neurotransmission in the brain.

## Materials and Methods

### Generation of *Gpr3^−/−^* mice and animal handling

The generation of *Gpr3^−/−^* mice and their genotyping by polymerase chain reaction (PCR) amplification of tail DNA were described previously [7]. Heterozygous progeny were backcrossed with CD1 females for 12–17 generations before generating the *Gpr3^−/−^* and *Gpr3^+/+^* founders. Experimental *Gpr3^−/−^* and *Gpr3^+/+^* mice were generated by a syngenic cross between founders.

All experiments were performed under blind conditions. Knockout and wild-type control males (25–30 g) were housed five per cage in temperature- (21±1°C) and humidity- (55±10%) controlled rooms with a 12-h light/12-h dark cycle (light between 8:00 AM and 8:00 PM). Food and water were available *ad libitum* during all experiments. Animal procedures were conducted in accordance with the guidelines of the European Communities, Directive 86/609/EEC regulating animal research and approved by the local ethical committee (CEEA-PRBB).

### Drugs

Fluoxetine hydrochloride (Sigma Chemical Co, Madrid, Spain) was dissolved in distillate water and diazepam in saline (0.09%). Both compounds were administered by intraperitoneal (i.p.) route 30 min before the test. The volume of injection was 0.1 ml per 10 g body weight. Control animals received the corresponding vehicle.

### Reverse transcription – polymerase chain reaction

Poly(A)+ RNA was extracted from tissues using the Fast Tract RNA purification kit (Invitrogen, Carlsbad, CA) and was reverse transcribed with Superscript II (Invitrogen, Carlsbad, CA) using oligo (dT) 12–18 primers. Primers as well as PCR reactions parameters were as shown in Table S1.

### *In situ* RNA hybridization

Digoxigenin-labeled RNA probe for GPR3 was obtained by reverse transcription of mouse genomic DNA corresponding to nucleotides 843–1491 (GenBank accession number D21062). After fixation by cardiac perfusion with 4% paraformaldehyde in PBS, brains were cryoprotected in sucrose 30%, frozen on dry ice, and 20 µm cryosections were cut and mounted on superfrost slides. *In situ* hybridization was performed as described previously [16].

### Behavioral responses

All experiments were conducted during the light cycle with naive male mice between 2 and 6 months of age. Animals were previously habituated to the testing rooms and handled for one week prior starting the experiments.

#### Locomotor activity

Mice were placed individually in locomotor activity boxes, consisting of a plastic rectangular area (9 cm×20×11 cm) with two crossed photocells, isolated in a soundproof furniture and in almost complete darkness (<5 lux) (Imetronic, Lyon, France). The number of activity counts was evaluated during 60 min on the testing day starting at 10.00 AM.

#### Open-field test

This method was used to evaluate locomotion under stressful situation. Briefly, each animal was placed in the open-field apparatus consisting of a rectangular area (70 cm wide×90 cm long×60 cm high) under bright illumination (500 lux). Sixty-three squares (10 cm×10 cm) were drawn with black lines on the floor of the apparatus. The parameters were measured during observation sessions of 5 min for three consecutive days. Two objects were placed in the field (a marble, 5.5 cm high, and a plastic figure, 4.5 cm high) exclusively on day 1 and the time spent to explore these two objects was recorded. Experiments were performed from 9.00 AM to 2:00 PM.

#### Skill learning and motor coordination on rota-rod

Animals were placed gently onto a rod of the rota-rod device. The speed of the rod was accelerated from 4 RPM to 20 RPM (acceleration 1 RPM/seconds) and kept constant until the end of the trial. We registered the time until the animal fell down. The cut-off time was 90 seconds. The animals were tested until they showed no further improvement in the performance, which means the variation between three consecutive trials was less than 30%. We expressed the speed of learning as the number of trials (with 95% confidence intervals, CI) necessary to reach 50% of the maximal performance.

#### Elevated plus-maze

This apparatus consisted of a plastic maze with four arms (16 cm long×5 cm wide) set in cross from a neutral central square (5 cm×5 cm). Two opposite arms were delimited by vertical walls (closed arms) whereas the two opposite arms had unprotected edges (open arms). The maze was elevated 30 cm above the ground and placed in indirect light (100 lux). Each mouse was placed in the central zone facing one of the open arms and was observed during 5 min for three consecutive days. An arm visit was recorded when the mouse moved all four paws into the arm. The total number of visits into the closed arms, open arms and the cumulative time spent in the open arms and closed arms were registered through a video camera system (Viewpoint, Lyon, France). The percentage of time spent and the percentage of entries in the open arms were then calculated for each animal. In a second experiment the anxiolytic effect of diazepam was evaluated in a group of naive mice. Diazepam was administered by i.p. route, 30 min before the test. The test was conducted exactly as described above.

#### Resident-intruder procedure

In this model, resident and intruder mice were put together for a period of 4 min. The attack score was calculated by registering the presence of an attack or menace (beating tail) by the resident for each 10-s period during the total 4-min period. The latency time (s) for the first attack was also registered. Resident mice were housed individually for 10 weeks prior to the beginning of the experimental procedure. Intruder animals were housed five per cage. Intruder and resident animals were carefully matched in age and weight. Animals received two training sessions in the morning and two tests sessions in the afternoon [17].

#### Forced swim test

This model was performed as previously reported [18]. Each mouse was placed in a transparent plastic cylinder filled with water (22±1°C) and submitted to a forced swim during 6 min. The total duration of immobility, including small maintenance movements, was measured during the last 4 min of the test.

#### Tail suspension test

A modified version of the previously described [19] was used in our experiments. Mice were individually suspended by the tail to a horizontal ring-star bar (distance from floor was 35 cm) using adhesive tape (distance from tip of tail was 2 cm). The recorded parameter was the cumulative number of seconds spent immobile during a total time of 6 min.

#### Active avoidance procedure

Mice were trained to avoid an aversive unconditioned stimulus (US) associated with a conditioned stimulus (CS, light) in a two-way shuttle box apparatus [20]. The US was an electric shock (0.2 mA) continuously applied to the grid of the floor. The apparatus (Panlab, Barcelona, Spain) consisted of a box with two compartments (20×10 cm) connected by a door. The CS preceded by 5 s the onset of the US and overlapped it for 25 s. By using this procedure, the light was presented in the compartment for 30 s, and at the end of this period, both CS and US were turned off. A conditioned response was recorded when the animal avoided the US by leaving the compartment within the 5 s after the onset of the CS. There was an inter-trial interval of 30 s between each trial session. Mice were subjected to once daily 100-trial active avoidance sessions during 5 consecutive days. A learning index was calculated for each animal and session by the ratio between the number of conditioned changes and the number of inter-trial changes of compartment to avoid potential influence of locomotor activity. The basal reactivity to electric foot-shock exposure was evaluated in a preliminary experiment in a separate group of *Gpr3^−/−^* and *Gpr3^+/+^* mice in order to avoid biased responses due to changes of locomotion and/or nociceptive sensitivity.

### Corticosterone levels measurement

Serum corticosterone levels from blood collected after decapitation, were measured by using the Coat-A-Count® Rat Corticosterone (Diagnostic Product Corporation, Los Angeles, CA, USA) as described previously [21].

### HPLC determination of endogenous catecholamine and indolamine content

Levels of monoamine neurotransmitters and their metabolites 3-methoxytyramine (3-MT), homovanillic acid (HVA), normetanephrine (NM) and 5-hydroxy indolacetic acid (5-HIAA) were determined by high performance liquid chromatography (HPLC) coupled with electrochemical and ultraviolet detection, according to the method described elsewhere [22]. More detailed information is provided in Materials and Methods S1.

### Construction of N terminus Rhodopsin-Tagged receptors

The addition of a tag corresponding to the first 19 amino acids of bovine rhodopsin at the amino terminus of the receptors allows assessment of surface expression with a tag-specific monoclonal antibody. The coding region of human *Gpr3* (NM005281: 96–1096) was swapped with the EcoRI - BamHI I486F hTSH fragment in SP-RT-I486F hTSH- pcDNA3 plasmid previously described in [23].

### Transfection Experiments

Chinese Hamster ovary (CHO-K1) cells cultured in Ham's F12 medium (Invitrogen, Merelbeke, Belgium), were used for transient expression experiments. They were transfected using the Fugene 6 reagent method (Roche, Indiapolis, IN) according to the manufacturer's instructions. Briefly, 2 10^6^ cells were seeded in 10-cm Petri dishes at day 1 and transfected with 5.5 µg ADN at day 2. At day 3, cells were detached by trypsinization and seeded in 24-well (2 10^5^/well) and 96-well plates (5 10^4^/well). At day 4, cells were used for flow immunofluorometry (24-well) and cAMP determinations (96-well). To determine the intracellular cAMP, medium was replenished with 25 µl of fresh culture medium containing 25 µM rolipram (Sigma, Bornem, Belgium) for 30 min. The reaction was terminated by adding 25 µl of lysis buffer. Quantitative cAMP determinations were made using the cAMP femto 2 bulk HTRF kit (Cisbio, Bagnols, France) according to manufacturer's manual. Triplicate wells were used for each assay. Non-transfected cells and cells transfected with the empty pcDNA3 were always run as negative controls. To find a linear relation between the number of receptors present at the cell surface and the cAMP accumulation, variable amounts of SP-RT hGpr3-pcDNA3 or SP-RT I486F hTSH-pcDNA3 constructs, completed with pcDNA3 vector to the same final DNA quantity, were transfected. SP-RT I486F hTSH is a mutant hTSH receptor endowed with strong constitutive activity. This linearity as well as a similar level of expression for both receptors were achieved when transfecting 0.1% of GPR3 or 1% of I486F TSH receptors in vector alone. The experiments were reproduced five times.

Sphingosine-1-phosphate (Biomol, Plymouth Meeting, PA) was prepared following the manufacturer's instructions and used at a final concentration of 10 µM in 0.032% fatty acid free Bovine Serum Albumine (Sigma, Bornem, Belgium) in Ham's F12 medium (mock).

### Quantification of Cell Surface Expression of GPR3 by Flow Immunocytometry

After detachment the cells were centrifuged at 500×*g* for 3 min at 4°C and the supernatant was discarded. They were then incubated for 30 min at room temperature in 100 µl PBS-BSA 0.1% containing the OR2-15A-6 monoclonal antibody directed against the N-terminus of bovine rhodopsin [24]. Cells were then washed with 2 ml PBS-BSA 0.1% and centrifuged as described above. They were incubated on ice in the dark for 30 min with fluoresceine-conjugated 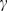-chain-specific goat antimouse IgG (Sigma, St Louis, MO) in the same buffer. Propidium iodide (10 µg/ml) was used for detection of damaged cells that were excluded from the analysis. Cells were washed as described above and resuspended in 250 µl PBS-BSA 0.1%. The fluorescence of 10,000 cells per tube was assayed by a FACScan Flow cytofluorometer (Becton Dickinson, Eerenbodegem, Belgium).

### Hippocampal cell culture and cAMP measurement

The results reported below were collected from five different litters, each experiment was made with *Gpr3* ^+/+^ and *Gpr3* ^−/−^ newborn mice of both sexes, born and prepared for culture on the same day. *Gpr3* ^+/+^ and *Gpr3* ^−/−^ mice were decapitated on the day of birth. Their brains were removed and placed in chilled (4°C) Leibovitz L15 medium enriched with 0.6% glucose. Tissue was mechanically dissociated with a fire-polished pipette into individual cells. Cells were collected by centrifugation, resuspended in Neurobasal-A supplemented with B27 and N2 (Invitrogen, Merelbeke, Belgium). 10^6^ cells from each hippocampus were plated in duplicate into a well of a 24-well plate precoated with Poly-D-lysine (Becton Dickinson, Erembodegem, Belgium) and maintained in a humidified incubator at 37°C with 95% atmospheric air/5% CO2. To determine the intracellular cAMP, 24 h after, medium was replenished with fresh culture medium containing 25 µM rolipram (Sigma, Bornem, Belgium) for 30 min. The reaction was terminated by aspirating the medium, adding 500 µl of 80% ethanol and placing at room temperature for 15 min. The extracts were lyophilized and reconstituted for cAMP level measurement using the cAMP femto 2 bulk HTRF kit (Cisbio, Bagnols, France) according to manufacturer's manual. Cultures were made in duplicate for each hippocampus. Results are calculated as the mean of minimum two dilutions in the linear part of the standard curve. Immunochemistry is described in Materials and Methods S1.

### Statistical Analysis

Data from behavioral studies were analyzed using a two-way Anova with repeated measures for the experiments on open-field test, elevated plus maze and the active avoidance paradigm. For these studies the factors of variation were genotype (between subjects) and day (within subjects). For the tail suspension test and forced swimming test and the evaluation of the diazepam effects on the elevated plus maze, two- way Anova were calculated. The factors of variation were genotype (between subjects) and treatment (between subjects). Corticosterone assay was analyzed by calculating a two-way Anova with genotype (between subjects) and stress exposure (between subjects) as variables. One-way Anova was calculated for genotype effect for data from intruder test as well as horizontal and vertical locomotor activities. Subsequent one-way Anova were calculated when required. Data from monoamine measurements were analyzed by using the Student t-test and cAMP levels with the Mann-Withney test. The level of significance was *p*<0.05.

## Results

### *Gpr3* ^−/−^ mouse model

**Figure 1:**
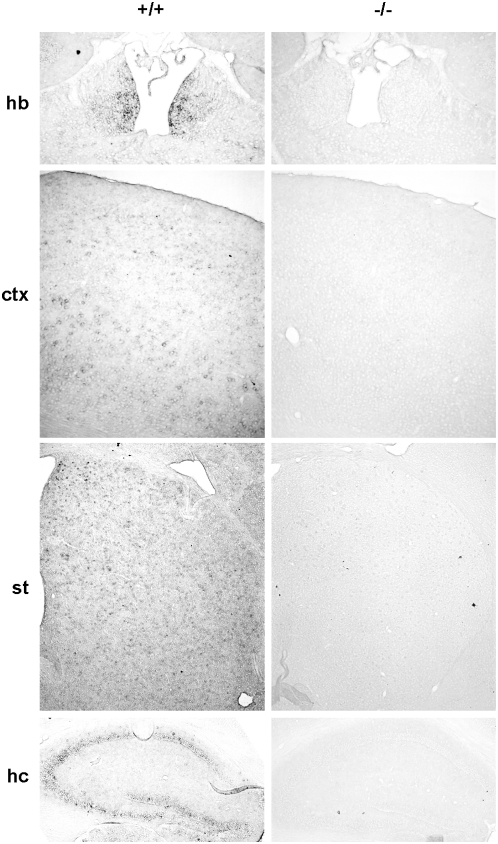
Expression of GPR3 in adult brain. *In situ* hybridization performed on *Gpr3^+/+^* and *Gpr3^−/−^* adult brain coronal sections, showing selective expression of *Gpr3* in the habenula (hb), and diffuse expression throughout the cerebral cortex (ctx), striatum (st), and layers of pyramidal cells in the hippocampus (hc). No hybridization signal could be detected in any brain region of *Gpr3^−/−^* mice.

To investigate the role of GPR3 in the brain we generated a mutant mouse line (*Gpr3^−/−^*) with a deletion encompassing the entire coding sequence of *Gpr3*. In the course of the present study mutant females were found to display important problems of fertility, which were described in detail elsewhere [7]. However, *Gpr3* ^−/−^ mice were viable and exhibited neither obvious developmental defects nor gross neurological alterations. The absence of *Gpr3* transcripts in *Gpr3* ^−/−^ mice was confirmed by reverse transcription (RT) polymerase chain reaction (PCR) on adult brains (see Figure S1). In agreement with previous studies [25] we found, by *in situ* hybridization, that GPR3 is widely expressed in several brain areas, with the highest levels in the habenula. *Gpr3* was also expressed diffusely throughout the cerebral cortex (in particular in sub- and supragranular layers), the striatum, and the hippocampus (including CA1, CA3 and dentate gyrus) (Figure 1).

### Increase in anxiety-like and despair-like behaviors, reaction to stress and aggressiveness in *Gpr3^−/−^* mice

**Figure 2:**
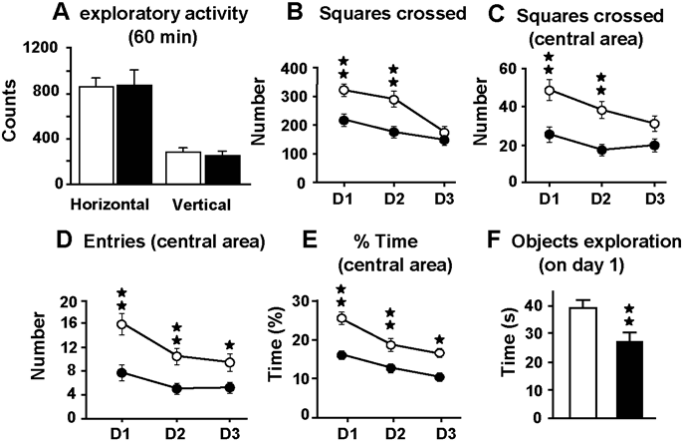
Spontaneous exploratory behavior in *Gpr3^−/−^* mice. Spontaneous horizontal and vertical locomotor activity under non-stressful conditions in both genotypes (*n Gpr3^−/−^* = 10; *n Gpr3^+/+^* = 10) (A). Locomotor activity in the open-field apparatus under stressful conditions (*Gpr3^−/−^ n* = 17; *Gpr3^+/+^ n* = 18) (B–F). Different parameters were observed during a 5 min-session: number of squares crossed (B), squares crossed in the central area (C), entries to the central area of the field (D), percentage of time spent in the central area (E) and the time spent exploring objects placed in the middle of the field on day one (F). Open bars and circles represent *Gpr3^+/+^*; black bars and circles represent *Gpr3^−/−^* mice. Black stars, genotype comparison. One star, *p*<0.05; two stars, *p*<0.01 (one-way ANOVA).

**Figure 3:**
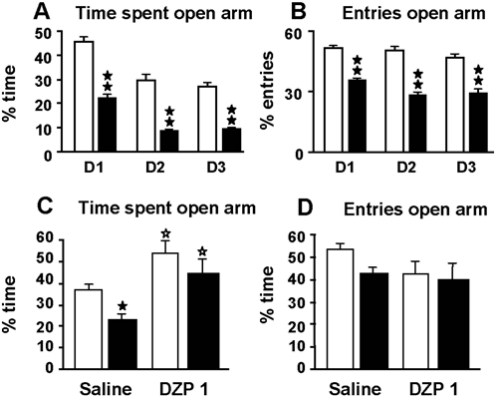
Increased anxiety-like responses in *Gpr3^−/−^* mice are reversed by diazepam. *Gpr3^−/−^* and *Gpr3^+/+^* mice were evaluated in the elevated plus-maze test. Animals were tested on three consecutive days (D1, D2 and D3; *Gpr3^−/−^ n* = 32; *Gpr3^+/+^ n* = 34) (A, B). The parameters evaluated were the percentage of time spent in the open arms (A) and the percentage of entries in the open arms (B). Open bars represent *Gpr3^+/+^*; black bars represent *Gpr3^−/−^* mice. The anxiety-like responses in the elevated-plus maze were measured after administration of vehicle or diazepam (1 mg/kg), 30 min before the test (*Gpr3^−/−^ n* = 10–12; *Gpr3^+/+^ n* = 9–11) (C, D). Black stars, genotype comparison (one-way ANOVA); white star, treatment comparisons (one-way ANOVA). One star, *p*<0.05; two stars, *p*<0.01.

**Figure 4:**
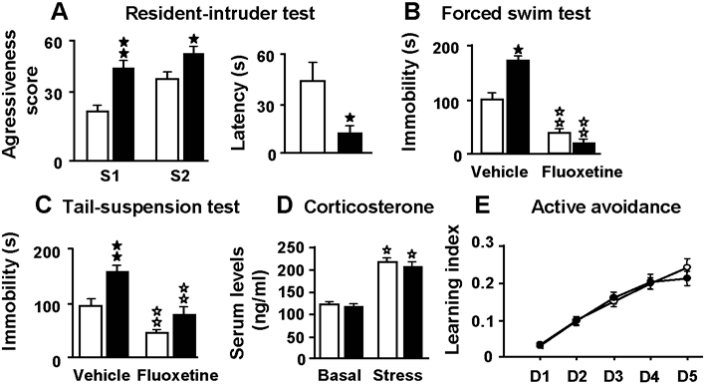
Increased despair responses in *Gpr3^−/−^* mice. *Gpr3^−/−^* and *Gpr3^+/+^* mice were evaluated in different behavioral paradigms. For the resident-intruder test, an aggressiveness score was calculated in the two first sessions (S1 and S2) and the latency period (s) of the first attack was registered (*Gpr3^−/−^ n* = 12; *Gpr3^+/+^ n* = 13) (A). The duration of immobility in the forced swim test (*Gpr3^−/−^ n* = 9; *Gpr3^+/+^ n* = 11) (B) and in the tail suspension test (*Gpr3^−/−^ n* = 12; *Gpr3^+/+^ n* = 13) (C) was measured after receiving vehicle or fluoxetine (10 mg/kg), 30 min before the test. Serum corticosterone levels (ng/ml) were measured under basal condition and after exposure to the tail suspension test (*n* = 9–10) (D). The learning performance was evaluated in the active avoidance paradigm on five consecutive days (D1 to D5; *Gpr3^−/−^ n* = 14; *Gpr3^+/+^ n* = 16). Learning index was calculated as the ratio between the conditioned responses and the inter-trial changes (E). Open bars and circles represent *Gpr3^+/+^*; black bars and circles represent *Gpr3^−/−^* mice. Black stars, genotype comparison (one-way ANOVA); white star, treatment comparisons (Scheffe's F-test). One star, *p*<0.05; two stars, *p*<0.01.

The effects of *Gpr3* inactivation on spontaneous behaviors and on reactions related to stress were analyzed. No difference between genotypes was found when exploratory activity was evaluated under non-stressful conditions (Figure 2A). Indeed, one-way Anova did not show genotype effect for horizontal nor vertical locomotor activities. However, under stressful conditions in the open-field test, *Gpr3^−/−^* mice displayed a decrease in exploratory behavior relative to wild-type on day 1 and day 2 of the test when the animals were not yet habituated to the environment. The number of squares crossed in the arena was lower in *Gpr3^−/−^* mice (Figure 2B). Two-way Anova showed a significant effect of the day (F_(2, 68)_ = 40.021, *p*<0.01); the genotype (F_(1, 34)_ = 9.366, *p*<0.01) and interaction between these two variables (F_(2, 68)_ = 7.848, *p*<0.01). In addition, the number of squared crossed in the central area of the open-field was lower for the *Gpr3^−/−^* mice [Figure 2C; day effect, (F_(2, 68)_ = 7.956, *p*<0.01); genotype effect (F_(1, 34)_ = 168.518, *p*<0.01), with no significant interaction between these factors]. The number of entries in the center of the field was also lower for the mutant mice [Figure 2D; day effect, (F_(2, 68)_ = 14,123, *p*<0.01); genotype effect, (F_(1, 34)_ = 128.071, *p*<0.01, without significant interaction between these two variables]. In agreement, the percentage of time spent in the central area was significantly lower in the *Gpr3^−/−^* mice on days 1, 2 and 3 [Figure 2E; genotype effect (F_(1, 34)_ = 162.1, *p*<0.01), and day effect (F_(2, 68)_ = 8.824, *p*<0.001) without interaction between these two factors]. Mice also exhibited a decreased exploration of unknown objects (F_(1, 34)_ = 8.104, *p*<0.01), thereby showing a clear neophobia and thigmotaxis (Figure 2F). Together these results suggested that *Gpr3^−/−^* mice displayed increased behavioral inhibition in a stressful novel environment, indicating an altered response to stressful events and/or an anxiety-like phenotype. The observed changes were not due to impaired motor coordination or altered skill tasks learning in *Gpr3^−/−^* mice since both genotypes exhibited similar performance in the rota-rod test (*Gpr3^+/+^* 8.20 trials, CI 6.037–10.363; *Gpr3^−/−^* 9.00 trials, CI 6.108–11.892). Half maximal performance was reached after similar number of trials in *Gpr3^+/+^* and *Gpr3^−/−^* animals, indicating that both genotypes had similar speed of learning. The anxiogenic-like phenotype was confirmed by the results obtained in the elevated plus-maze (Figure 3A, B). In this test, mice face a conflict between an aversion to the open arms of the maze and the motivation to explore them. *Gpr3^−/−^* mice displayed a decrease in the percentage of entries and time spent in the open arms of the maze on the three observation days (Figure 3A, B). Indeed two-way Anova calculated for the time spent in the open arm showed a significant effect of the day (F_(2, 128)_ = 73.441, *p*<0.01) and the genotype (F_(1, 64)_ = 82.374, *p*<0.01) without interaction between these two factors. In the same way, two-way Anova for the entries in the open arm detected a significant effect of the day (F_(2, 128)_ = 8.194, *p*<0.01) and the genotype (F_(1, 64)_ = 109.185 *p*<0.01) but no significant interaction emerged. The anxiety-like responses observed in the elevated plus-maze were similarly reversed by diazepam administration (1 mg/kg) in both genotypes. Indeed two-way Anova calculated for the time spent in the open arm showed a significant effect for the genotype (F_(1, 38)_ = 8.074, *p*<0.01) and treatment (F_(1, 38)_ = 8.208, *p*<0.01) without interaction between these two factors (Figure 3C). In addition, at the dose used (1 mg/kg, ip), diazepam did not induce significant changes in locomotor activity since the number of entries in the open arm was not significantly different in animals treated with the benzodiazepine (Figure 3D). To investigate whether their anxiety-like behavior was associated with aggressiveness, we evaluated *Gpr3^−/−^* mice in the resident-intruder test. *Gpr3^−/−^* resident male mice exhibited a higher aggressiveness towards intruders than wild-type mice as estimated by their higher aggressiveness score in two sessions (session 1, F_(1, 20)_ = 4.016, *p*<0.01; session 2 (F_(1, 19)_ = 4.016, *p*<0.05), and their shorter latency period for the first attack (F_(1, 20)_ = 4.501, *p*<0.05) (Figure 4A). We next examined the *Gpr3^−/−^* profiles in two different behavioral paradigms widely used to assess behavioral traits that may be related to despair behavior, the forced swim and the tail suspension tests. Both tests are based on the assumption that mice will normally try to escape from an inescapable aversive stimulus. An enhanced immobility is thought to represent a state of behavioral despair that is characteristic of depression [26], [27]. *Gpr3^−/−^* mice exhibited an increase in the duration of immobility compared with wild-type mice in both behavioral models. As expected, administration of the inhibitor of serotonin reuptake fluoxetine (10 mg/kg, i.p., 30 min before the test) reduced the period of immobility of wild-type mice in both tests ([27]; Figure 4B, C). *Gpr3^−/−^* mice appeared to be sensitive to this dose of fluoxetine since its administration abolished statistical difference between genotypes in both tests. Two-way Anova for the forced swim test showed a significant effect for treatment (F_(1, 39)_ = 175.05, *p*<0.01) and interaction between treatment and genotype (F_(1, 39)_ = 10.497, *p*<0.01) but no genotype effect. For the tail suspension, two-way Anova revealed a significant effect for treatment (F_(1, 39)_ = 16.254, *p*<0.001) and genotype (F_(1, 39)_ = 11.255, *p*<0.01) but no significant interaction emerged. Since dysregulation of the hypothalamic-pituitary adrenal (HPA) axis can lead to the onset of anxiety-like behavior and depression, we compared in *Gpr3^−/−^* and wild-type mice the levels of serum corticosterone, the major glucocorticoid end product in rodents, both under basal conditions and in response to the stress induced by the tail suspension test. The basal corticosterone levels were comparable in both genotypes (Figure 4D). In addition, corticosteronelevels were similarly increased after stress despite the significant increase in the duration of immobility observed for *Gpr3^−/−^* mice (Figure 4D). Two-way Anova calculated for genotype and stress exposure revealed a stress effect (F_(1, 38)_ = 14.489, *p*<0.01) with neither genotype effect nor interaction between the two factors. We next investigated if the altered emotional-like state exhibited by the knockouts, could alter their learning ability in an active avoidance paradigm. In this task the mouse has to learn to avoid an aversive event in response to a stimulus cue, by actively moving to a different compartment. The electric foot-shock used as aversive stimulus was found to produce similar basal reactivity in both genotypes as evaluated, in a preliminary experiment, by the changes of compartment, locomotor activity, jumps and vocalizations (data not shown). *Gpr3^−/−^* and *Gpr3^+/+^* exhibited a similar progressive increase in the avoidance learning performance through the five days of training as assessed by the significant increase in the number of conditioned changes in both genotypes (Figure 4E). Two-way Anova revealed a significant effect for the day (F_(4, 112)_ = 82, 602, *p*<0.01) but neither genotype effect nor interaction between the two variables. Therefore, the lack of GPR3 does not seem to prevent the knockouts from adapting themselves to adverse conditions in this paradigm.

### Monoamine neurotransmitter levels are altered in multiple brain areas in *Gpr3^−/−^* mice

**Figure 5:**
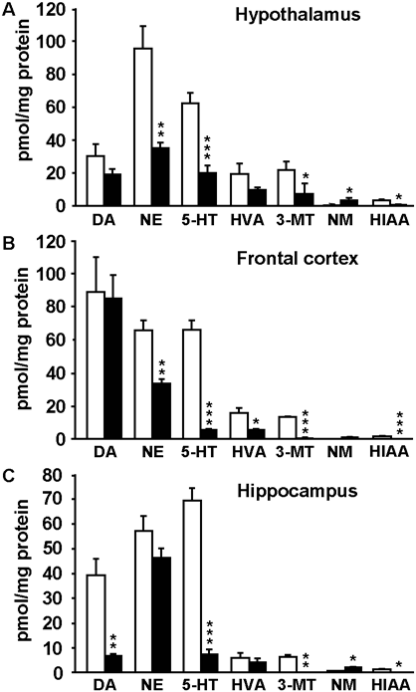
Alteration in monoamine neurotransmitter levels in multiple areas in *Gpr3^−/−^* mice. Tissue content of dopamine (DA), noradrenaline, (NE), and serotonin, (5-HT) and their metabolites 3-methoxytyramine (3-MT), homovanillic acid (HVA), normetanephrine (NM) and 5-hydroxy indolacetic acid (HIAA) were analyzed by HPLC in the hypothalamus (A), frontal cortex (B), and hippocampus (C) of *Gpr3^+/+^* (opens bars) and *Gpr3^−/−^* mice (black bars). The results are expressed in pmol/mg protein. Stars indicate significant differences between *Gpr3^+/+^* and *Gpr3^−/−^* mice calculated by the Student t-test (One star *p*<0.05, two stars *p*<0.01, three stars *p*<0.001, *Gpr3^−/−^ n* = 5; *Gpr3^+/+^ n* = 6).

Given the high expression of *Gpr3* in the habenula, which is involved in the monoamine regulation [12], we determined whether differences in monoaminergic neurotransmission may underline the behavioral alterations in *Gpr3^−/−^* mice. We measured levels of dopamine (DA), noradrenaline (NE) and serotonine (5-HT) and their metabolites 3-methoxytyramine (3-MT), homovanillic acid (HVA), normetanephrine (NM) and 5-hydroxy indolacetic acid (HIAA) in the amygdala, hippocampus, hypothalamus, striatum and frontal cortex in knockout and wild-type littermates. 5-HT and NE were significantly reduced in the hypothalamus and frontal cortex (Figure 5A, B). DA and 5-HT were also found dramatically reduced in the hippocampus (Figure 5C). Among the metabolites, HIAA, the major metabolite of 5-HT and 3-MT, the primary metabolite of DA were significantly reduced, whereas NM, the primary metabolite of NE was elevated in all three regions (Figure 5A, B, C). In addition HVA, the secondary metabolite of DA was also significantly reduced in the frontal cortex (Figure 5B). No differences between genotypes were found in striatum and amygdala (not shown). These changes in monoamine neurotransmitters content could support the behavioral alterations observed in *Gpr3^−/−^* mice.

### Inability of sphingosine 1-P to affect GPR3 constitutive basal activity

In an attempt to link the phenotype of *Gpr3^−/−^* animals with the regulation of GPR3 activity, we re-explored the role of sphingosine 1-P, previously proposed as a possible agonist of the receptor [11]. We measured the cAMP accumulation induced by transient expression of a *Gpr3* plasmid in CHO cells in basal conditions and in presence of 10 µM sphingosine 1-P. cAMP levels were compared with those of the highly constitutively active I486F TSH receptor mutant [23]. In order to compare cAMP accumulation at similar level of expression for both receptors and under conditions in which cAMP accumulation is proportional to expression, we transfected 0.1% of GPR3 or 1% of I486F TSH receptor constructs diluted in the empty vector.

**Figure 6:**
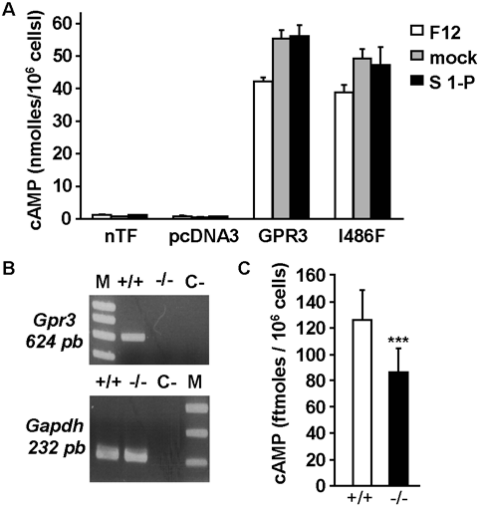
Adenylyl cyclase-stimulation properties of GPR3. (A) A representative experiment is shown. Bars correspond to cAMP accumulation in CHO cells transfected or not (nTF) with the empty vector (pcDNA3), GPR3 or I486F TSH (I486F) plasmids and cultured in serum free medium (F12). The effect of 10 µM S1P (S 1-P) has to be compared with mock medium (mock). (B) RT-PCR detection of *Gpr3* mRNA in primary hippocampal culture of newborn *Gpr3^+/+^* (+/+) and *Gpr3^−/−^* (−/−) mice. Water was added instead of sample to test for contamination with extraneous DNA (C-). *Gapdh* (a house keeping gene) was amplified simultaneously in each cDNA preparation as internal control. (C) Bars correspond to cAMP accumulation in primary hippocampal culture of newborn *Gpr3^+/+^* (+/+) and *Gpr3^−/−^* mice (−/−). cAMP levels are expressed in fmoles / 10^6^ cells plated 24 h before measurements. Stars indicate significant differences between *Gpr3^+/+^* and *Gpr3^−/−^* mice calculated by the Mann-Withney test (three stars *p*<0.001, *n* = 20).

As expected, the expression of GPR3 resulted in a dramatic stimulation of cAMP accumulation. Noteworthy, this effect was observed in cells cultured in serum-free medium. The stimulation amplitude was comparable to that observed with the I486F TSH receptor when normalized to surface expression (Figure 6A).

cAMP accumulation in mock condition (0.032% fatty acid free Bovine Serum Albumine in serum free Ham's F12 medium) was unaffected after a one-hour incubation with 10 µM sphingosine 1-P (Figure 6A) thereby refuting, in our hands, its previously described agonist properties [11].

### Decrease of intracellular cAMP levels in primary hippocampal culture of *Gpr3^−/−^* mice

Although the consequence of the absence of GPR3 on fertility was shown to be directly correlated with a decrease in oocyte cAMP [9], [10], there are no data connecting endogenous GPR3 expression with cAMP production in the CNS. Therefore, we decided to evaluate modifications of cAMP levels in the brain. For this purpose, we measured the basal cAMP levels in primary cultures of newborn *Gpr3^+/+^* and *Gpr3^−/−^* hippocampal neurons 24 h after plating. RT PCR experiments confirmed the *Gpr3* expression in *Gpr3^+/+^* culture (Figure 6B). Basal cAMP levels were found decreased in *Gpr3^−/−^* mice compared with wild-type (*p*<0.001; Figure 6C). The percentages of neuronal cells were evaluated by the number of cells immunostained using the antibodies TUJ1 directed against β-tubulin neuronal isoform III and Ctip2, a transcriptional repressor that is highly expressed in hippocampal neurons. They did not differ between *Gpr3^+/+^* and *Gpr3^−/−^* cultures (see Figure S2). The presence of few glial cell clusters immunostained with an antibody directed against GFAP (glial fibrillary acidic protein), a marker of astrocytes, was similar in both genotypes (data not shown). These data indicate an alteration of cAMP signaling in the CNS of *Gpr3^−/−^*, providing the first potential link between the receptor constitutive activity and the behavioral phenotype.

## Discussion

The orphan receptor GPR3 is a G protein-coupled receptor endowed with constitutive Gs signaling activity which is expressed both in peripheral tissues and the CNS [25]. In this study, we investigated the consequences of *Gpr3* deletion on several behavioral responses under the control of CNS, in particular the emotional-like responses.

*Gpr3^−/−^* mice developed and behaved normally with neither major changes in locomotion under basal conditions nor impairment in motor coordination. No deficits in avoidance learning were evidenced in *Gpr3^−/−^* mice, which exhibited similar performance than wild-type mice in the active avoidance paradigm. This suggests that the lack of GPR3 does not affect the learning of fear which primarily develops through a conditioned process. However, *Gpr3^−/−^* mice exhibited a higher level of anxiety-related responses after exposure to unfamiliar stressful environment in the open-field and the elevated plus-maze paradigms, including a behavioral inhibition observed as a reduced activity in the open-field test. The anxiety-like phenotype observed in the elevated plus-maze was sensitive to the effect of benzodiazepines since the administration of diazepam reversed the anxiogenic response. In addition, an increase in the number of attacks and a decrease in the latency period for the first attack were observed in the resident-intruder test, revealing a higher level of aggressiveness in mutant mice. Thus, the behavioral phenotype argues for a possible link between emotional reactivity and aggressive behavior in mutant mice [28]. It also suggests that *Gpr3^−/−^* mice could be more susceptible to develop a “depression-related” behavior when exposed to a stressful situation from which they cannot escape. Indeed *Gpr3^−/−^* mice exhibited a behavioral despair as evidenced by an increased duration of immobility in the tail suspension and the forced swim tests, which are widely used to assess the efficacy of antidepressant drugs and genetic manipulation relevant to depression. It was suggested that this acceptance of uncontrollable situation was analogous to the apathetic despair related to a depressive-like state [27]. We did not notice any change in serum corticosterone levels in *Gpr3^−/−^* mice under basal conditions or in response to the stress induced by the tail suspension test. Therefore, the mechanism underlying the behavioral characteristics of *Gpr3^−/−^* mice does not seem to be related to an alteration of the HPA axis activity. Previous findings revealed possible dissociation between behavioral responsiveness and HPA axis activity in various experimental conditions in rodents [29], [30], [31], [32].

Monoaminergic neurotransmission is thought to modulate mood states and the stress response. Thereby, important alterations of the brain monoaminergic systems have been involved in mood disorders [14], [15]. Furthermore, animal studies proposed an inverse relationship between the activity of the brain 5-HT system and aggressive behavior [33], [34], [35]. Therefore, we analyzed the tissue levels of 5-HT, NE and DA in several brain structures of *Gpr3^−/−^* mice. We found that *Gpr3^−/−^* mice exhibit abnormally low levels of monoamines in various brain areas under basal conditions. In particular, the dramatic decrease in 5-HT content observed in hippocampus, hypothalamus and frontal cortex could well account for the behavioral despair and aggressiveness displayed by the *Gpr3^−/−^* mice and indicate a role for GPR3 in modulating the serotonergic system. The observation of a significant decrease in NE in cortex and hypothalamus could also account for the behavioral phenotype of knockout mice. Because the metabolites of 5-HT and DA were also decreased in *Gpr3^−/−^* mice, it seems likely that the primary target of the regulation by GPR3 is the synthesis or reuptake of these neurotransmitters. Conversely, in case of NE, the primary metabolite NM was found to be increased indicating that its metabolism is affected by *Gpr3* deletion.

The expression of GPR3 showed the highest level in the habenula. The habenular complex is an evolutionarily conserved diencephalic structure linking the forebrain with midbrain and hindbrain structures [12], [13]. It has been shown to participate in the regulation of monoamine transmission [12]. Through a habenulo-raphe pathway it modulates serotonergic activity in many structures including the hippocampus and influences the noradrenergic activity through a connexion with the locus coeruleus [36], [37]. Our results support this statement and suggest that such a control is modulated at least in part through GPR3.

In numerous brain areas, changes in the activity of the cAMP pathway have been associated with behavioral changes [2], [3], [4]. Given the ability of GPR3 to activate the cAMP regulatory cascade in a tonic way, and in agreement with our observation of lower cAMP levels in primary cultured *Gpr3^−/−^* neurons, GPR3 effects could be mediated through the cAMP-response-element-binding protein (CREB). Indeed, the CREB transcription factor is known to mediate many effects of cAMP on gene expression and to play a crucial role in depression- and anxiety-related disorders [2], [3], [4]. CREB controls the efficacy of synaptic transmission by regulating the efficiency of neurotransmitter release from presynaptic terminals [3], [4]. The lack of GPR3 could possibly impact on the activity of the efferent connections of the habenular complex and account for the important alterations of the brain monoamines that we found in various brain areas of mutant mice.

Deletion of genes expressed in the medial habenula have been reported to elicit abnormal anxiety-related behavioral responses, suggesting that the medial habenula could participate in the anxiety phenotype observed in the mutant mice [38], [39], [40]. In addition, several animal models for despair behavior show an elevated metabolism in this brain structure [41], [42], [43], [44]. Taken together our study supports the hypothesis that lack of GPR3 impairs the monoaminergic modulatory function of habenula, thereby resulting in a reduced ability of *Gpr3* ^−/−^ mice to cope with stress.

Interestingly, mice deficient in phosphodiesterase-4D (PDE-4D) and animals treated with the PDE-4 inhibitor rolipram display an attenuated despair behavior when exposed to the same behavioral models used in the present study [5]. Since GPR3 and PDE-4D are both highly expressed in medial habenula and have antagonistic action on the intracellular cAMP level, these data strengthen the notion that basal levels of cAMP in habenular neurons are an important parameter for the emotional-like behaviors under the control of the limbic system [45].

In conclusion, this study demonstrates that GPR3 plays an important role in modulating several responses in animal models consistently employed to evaluate emotional disorders including anxiety, depression-like disorders, and aggressiveness, probably by tuning the monoaminergic neurotransmission in various brain regions. In consequence, GPR3 dysfunction could be involved in the etiology of disorders associated with emotional disturbances, thereby representing a novel actor of the cAMP-dependent signaling pathway linked to behavioral responses. Central to this question is the nature, if any, of the endogenous GPR3 agonist [46]. From an evolutionary viewpoint and its position in sequence similarity dendrograms, GPR3 belongs to a subfamily of receptors in which both peptide and lipid receptors are found [47] and the lipid sphingosine 1-P has been proposed as a natural GPR3 agonist [10], [11]. This has been however questioned [48] and could not be reproduced in the present study. The possibility must be considered that receptors with such a strong constitutive activity might be controlled by natural inverse agonists, or would have no endogenous ligand at all [6], [49], [50]. In this case, GPR3 receptors would be expected to control regulatory cascades in a tonic way via the adjustment of their expression level or via the regulation of their signaling efficacy [51], [52]. Identification of the endogenous ligand(s) of GPR3 or agents capable of modulating its constitutive activity is expected to open new therapeutic avenues in the treatments of disorders affecting mood or anxiety.

## Supporting Information

Materials and Methods S1 (0.03 MB DOC)

Table S1 (0.03 MB DOC)

Figure S1 Expression of GPR3 in adult brain. (A) RT-PCR detection of Gpr3 mRNA in total brains. As internal controls Gapdh (a house keeping gene) and Gpr19 (an orphan GPCR gene) were amplified simultaneously in each cDNA preparation. (B) Autoradiography of nitrocellulose transferred RT-PCR gel hybridized with specific 32P labelled probes specific for Gapdh, Gpr19 and Gpr3. The faint upper band visible in Gpr3−/− in panel A was not specific as assessed by the absence of hybridization with a Gpr3 probe in the present panel.
(0.55 MB TIF)

Figure S2 Primary hippocampal culture of Gpr3+/+ and Gpr3−/− newborn immunostained for Tuj1 and Ctip2, 24 h after plating. (A) Percentages of immunostained cells / cells with DAPI positive nuclei. Open bars represent Gpr3+/+; black bars represent Gpr3−/− mice (n = 8). (B–C) Double-label fluorescent immunohistochemistry showing Tuj1 (green) and Ctip2 (red) positive neurons in Gpr3−/− (B) and Gpr3+/+ (C) cultures.
(1.15 MB TIF)
