# Supplementary material for: GPR3 Receptor, a Novel Actor in the Emotional-Like Responses
Source: PLoS One. 2009 Mar 4;4(3):e4704. doi: 10.1371/journal.pone.0004704 (PMC2649507; doi:10.1371/journal.pone.0004704)
Supplement: Materials and Methods S1 — (0.03 MB DOC) [file pone.0004704.s001.doc]

**Supporting Materials and Methods.**

# HPLC determination of endogenous catecholamine and indolamine content.

Animals were sacrificed by decapitation. After the preparation of different brain areas the native tissue was frozen by liquid nitrogen. The weighed frozen tissue was homogenized in an appropriate volume of ice-cold 0.1 M perchloric acid that contained theophylline (as an internal standard) at 10 nmol/ml concentration and 0.5 mM sodium metabisulphite (antioxidant for biogenic amines). The suspension was centrifuged at 300 g (4500 rpm) for 10 min at 0-4^o^C. The perchloric anion was precipitated by addition of 3 µl of 1 M potassium hydroxide to 70 µl of the supernatant. The precipitate was then removed by centrifugation. The supernatant was kept at -20^o^C until analysis. The pellet was saved for protein measurement according to [53].

For the measurement of biogenic amines a liquid-liquid two-dimensional reversed-phase and ion pair-reversed-phase chromatographic separation was applied [22] using a Gilson liquid chromatographic System (Gilson Medical Electronics inc., Middletown, and WI USA) equipped with Applied Biosystems 785/A UV and BAS CC-4 amperometric detector in a cascade line. For sample cleaning a “trap-column” (15-25 µm Nucleosil C-18 (20x4.0) was inserted into a loop position. The separations of neurotransmitters were performed on a 3 µm Discovery C18 HS (150 x 4.0 mm) analytical column accomplished with ion pair-reversed-phase buffer at constant flow rate 0.8 ml min^-1^ from the 11^th^ to 55^th^ min of analysis. The detection of internal standard (theophilline) was performed at 254 nm wavelengths by UV and the biogenic amines at + 0.73 V potential of electrochemical detection. The retention order were as follows: noradrenaline (NE) 16.7; 5-hydroxy indolacetic acid (5-HIAA) 23.8; normetanephrine (NM) 25.0; homovanillic acid (HVA) 26.8; dopamine (DA) 28.5; 3-methoxytyramine (3-MT) 51.2; and 5-hydroxytryptamine (5-HT) 53.3 min. Concentrations were calculated by a two-point calibration curve internal standard method: (A_i_ * f * B)/(C * D_i_ * E) (A_i_: Area of biogenic amine component; B: Sample volume; C: Injection volume (45.5 or 22.7 µl); D_i_: Response factor of 1 pmol biogenic amine standard; E: Protein content of sample; f: factor of Internal Standard (IS area in calibration/IS area in actual). The data were expressed as pmol per mg protein.

**Immunochemistry**

Twenty-four hour after their plating on Poly-D-Lysine precoated 4-well CultureSlides (BD Biosciences, Belgium), the cells were rinsed in PBS and fixed by 4% paraformaldehyde in PBS for 30 min at 4°C. After 3 rinses in PBS, the cells were incubated with PBS containing 0.3% Triton X-100, 5% normal horse serum, 3% BSA for 30 min at room temperature. Following incubation with primary antibody (overnight at 4°C) in 1% normal horse serum, 3% BSA, 0.1% Triton X-100, the cells were rinsed three times in PBS and incubated with secondary antibodies in the same medium for 60 min in the dark. After three washes in PBS, cells were stained with DAPI as nuclear marker and then mounted in FluorSave (Calbiochem, La Jolla, CA). Fluorescence was detected and photographed with a photomicroscope Axio Imager M1 (Zeiss, Zaventem, Belgium). Percentages of stained cells were evaluated in cells showing non pycnotic DAPI positive nuclei. The following primary antibodies were used: Tuj1 MMS-435P (Covance, Berkeley, USA), Ctip2 ab18465 (Abcam, Cambridge, UK) and GFAP G9269 (Sigma, Saint Louis, USA).
