# Supplementary material for: GPR3 Receptor, a Novel Actor in the Emotional-Like Responses
Source: PLoS One. 2009 Mar 4;4(3):e4704. doi: 10.1371/journal.pone.0004704 (PMC2649507; doi:10.1371/journal.pone.0004704)
Supplement: Table S1 — (0.03 MB DOC) [file pone.0004704.s002.doc]

**Table S1. Primers used for PCR amplification of *Gpr3*, *Gapdh* and *Gpr19* cDNA.**

| DNA fragments | Name | Primer sequence | Amplified  fragment size (bp) |
| --- | --- | --- | --- |
| *Gapdh* | GAPDD1  GAPDR1 | For 5’-GGAGCCAAACGGGTCATCATCTC-3’  Rev 5’- GAGGGGCCATCCACAGTCTTCT-3’ | 232 |
| *Gpr19* | NGAD1  NGAR2 | For 5’-TCAGAACGGCAGCTGCGCGG-3’  Rev 5’-AGCAGGAACATCTTGACCGTT-3’ | 770 |
| *Gpr3* | 06RTD1  06RTR1 | 3’For 5’-TAGCTTGGCCGTAGCAGAC-3’  Rev 5’- TCATGGAGTTGTAGGTGGCT -3’ | 624 |

For RT-PCR, thermocycler parameters were as follows: denaturation (60 s at 94°C), annealing (60 s at 62°C for *Gapdh*, at 59°C for *Gpr3* and at 60°C for *Gpr19*), and extension (60 s at 72°C), 30 cycles. Reverse transcriptase was omitted from some samples to control for false-positive PCR amplification of contaminating genomic DNA. Water was added instead of sample to test for contamination with extraneous DNA.

PCR reaction products were analyzed by electrophoresis on 1.2% agarose gel stained with ethidium bromide to visualize products on a UV transilluminator. DNA was transferred from the gel to a nitrocellulose membrane which was hybridized with specific respective probes before exposed to X-ray film to obtain an autoradiographic image.
