# Supplementary figures and images for: GPR3 Receptor, a Novel Actor in the Emotional-Like Responses
Source: PLoS One. 2009 Mar 4;4(3):e4704. doi: 10.1371/journal.pone.0004704 (PMC2649507; doi:10.1371/journal.pone.0004704)

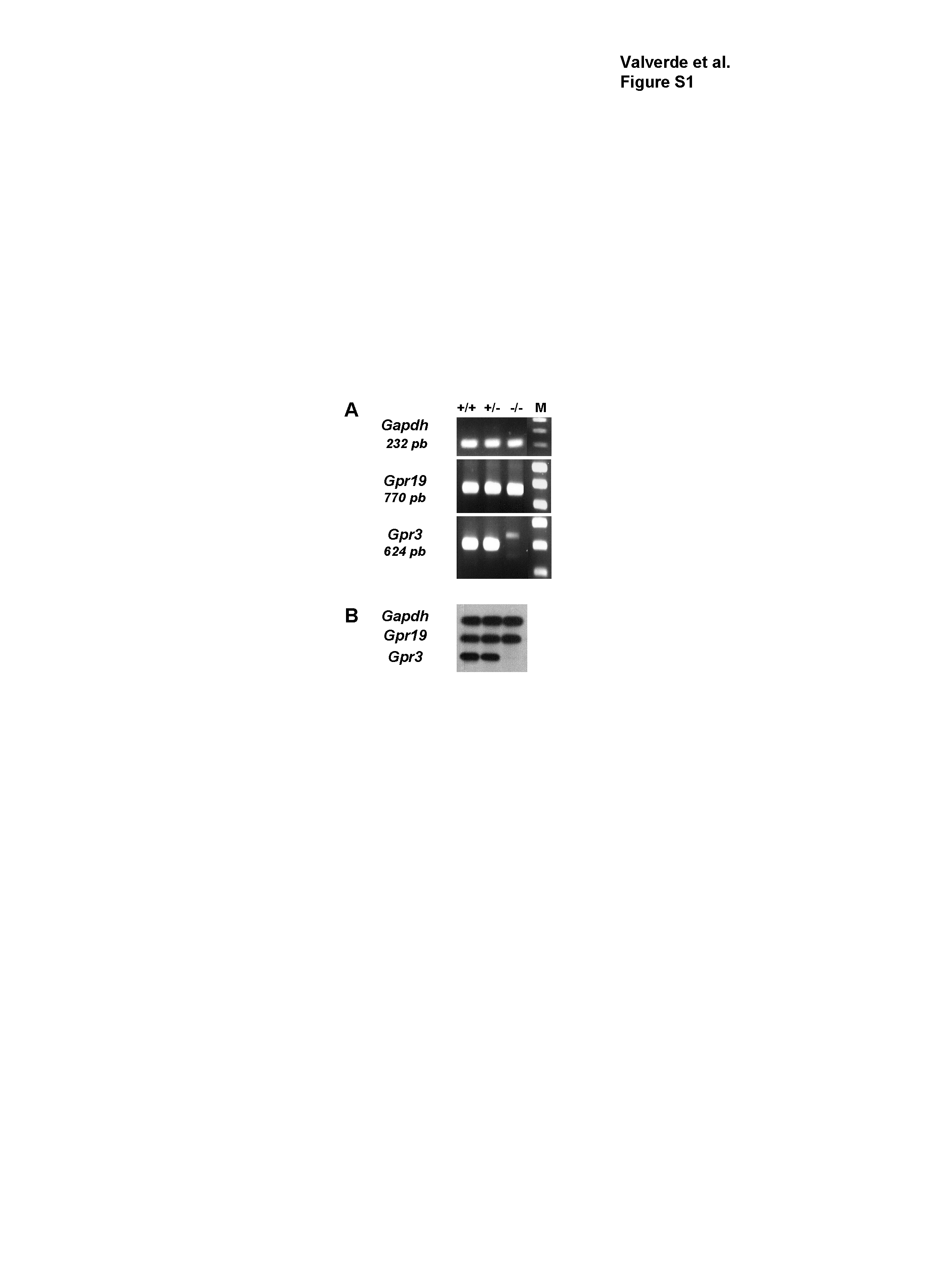

Supplement: Figure S1 — Expression of GPR3 in adult brain. (A) RT-PCR detection of Gpr3 mRNA in total brains. As internal controls Gapdh (a house keeping gene) and Gpr19 (an orphan GPCR gene) were amplified simultaneously in each cDNA preparation. (B) Autoradiography of nitrocellulose transferred RT-PCR gel hybridized with specific 32P labelled probes specific for Gapdh, Gpr19 and Gpr3. The faint upper band visible in Gpr3−/− in panel A was not specific as assessed by the absence of hybridization with a Gpr3 probe in the present panel. (0.55 MB TIF) [file pone.0004704.s003.tif]

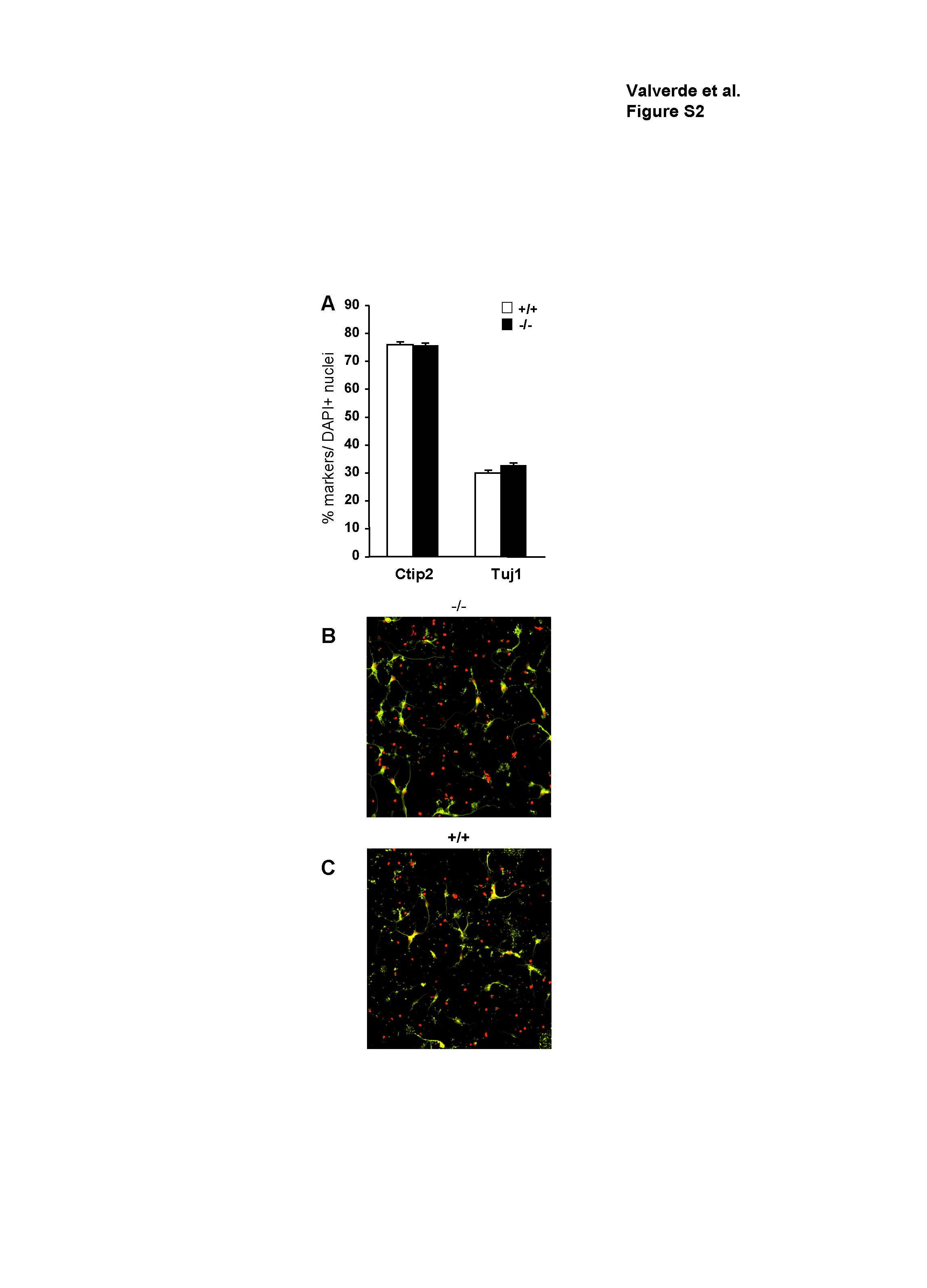

Supplement: Figure S2 — Primary hippocampal culture of Gpr3+/+ and Gpr3−/− newborn immunostained for Tuj1 and Ctip2, 24 h after plating. (A) Percentages of immunostained cells / cells with DAPI positive nuclei. Open bars represent Gpr3+/+; black bars represent Gpr3−/− mice (n = 8). (B–C) Double-label fluorescent immunohistochemistry showing Tuj1 (green) and Ctip2 (red) positive neurons in Gpr3−/− (B) and Gpr3+/+ (C) cultures. (1.15 MB TIF) [file pone.0004704.s004.tif]
